# Cytoplasmic tail determines the membrane trafficking and localization of SARS-CoV-2 spike protein

**DOI:** 10.3389/fmolb.2022.1004036

**Published:** 2022-09-26

**Authors:** Qinlin Li, Yihan Liu, Leiliang Zhang

**Affiliations:** ^1^ Shandong Provincial Hospital Affiliated to Shandong First Medical University, Jinan, China; ^2^ Department of Pathogen Biology, School of Clinical and Basic Medical Sciences, Shandong First Medical University & Shandong Academy of Medical Sciences, Jinan, China; ^3^ Medical Science and Technology Innovation Center, Shandong First Medical University and Shandong Academy of Medical Sciences, Jinan, China

**Keywords:** SARS-CoV-2, spike, COPI, SNX27, palmitoylation

## Abstract

The spike (S) glycoprotein of SARS-CoV-2 mediates viral entry through associating with ACE2 on host cells. Intracellular trafficking and palmitoylation of S protein are required for its function. The short cytoplasmic tail of S protein plays a key role in the intracellular trafficking, which contains the binding site for the host trafficking proteins such as COPI, COPII and SNX27. This cytoplasmic tail also contains the palmitoylation sites of S protein. Protein palmitoylation modification of S protein could be catalyzed by a family of zinc finger DHHC domain-containing protein palmitoyltransferases (ZDHHCs). The intracellular trafficking and membrane location facilitate surface expression of S protein and assembly of progeny virions. In this review, we summarize the function of S protein cytoplasmic tail in transportation and localization. S protein relies on intracellular trafficking pathways and palmitoylation modification to facilitate the life cycle of SARS-CoV-2, meanwhile it could interfere with the host transport pathways. The interplay between S protein and intracellular trafficking proteins could partially explain the acute symptoms or Long-COVID complications in multiple organs of COVID-19 patients.

## Introduction

Current pandemic of Corona Virus Disease 2019 (COVID-19) is caused by severe acute respiratory syndrome coronavirus 2 (SARS-CoV-2) (Hoffmann et al., 2020). Based on epidemiological studies, the clinical symptoms of COVID-19 range from asymptomatic to severe illness, and they vary over time. COVID-19 patients may experience numerous symptoms including fever, dry cough, shortness of breath, headache, new loss of taste or smell, sore throat and so on (Zhou et al., 2020). SARS-CoV-2 infection is not merely restricted to the respiratory system, but also could trigger an excessive immune response, contributing to multi-organ dysfunction or death (da Rosa Mesquita et al., 2021). More and more survivors were reported to experience persistence or appearance of diverse symptoms with varying degrees. They suffered a wide variety of postinfectious complications that last for weeks or even months after recovery from acute illness. Defined as Long-COVID by World Health Organization (WHO), these symptoms cover fatigue, shortness of breath, hypertension, diabetes and cognitive dysfunction, but also others and generally have an impact on everyday functioning (Yong and Liu, 2022). Long-COVID, affecting cardiovascular system, respiratory system, nervous system and so on, seems to be threatening to human gradually.

**FIGURE 1:**
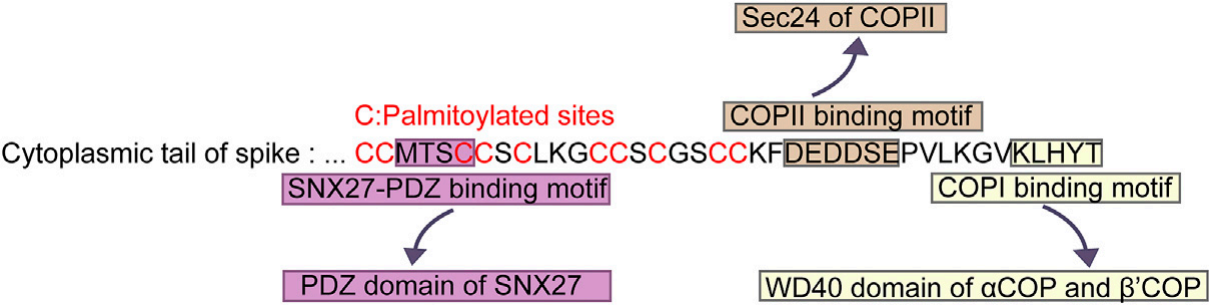
Key sequences in cytoplasmic tail of SARS-CoV-2 S protein for palmitoylation and association with transport proteins. “MTSC” binds to PDZ domain of SNX27. “DEDDSE” associates with Sec24 subunit of COPII. “KLHYT” interacts with WD40 domain of αCOP and β′COP.

SARS-CoV-2 is an enveloped virus encoding four structural proteins: the spike (S) protein mediating entry to the host cell, the nucleocapsid (N) protein encapsulating the viral RNA, the membrane (M) protein and the envelope (E) protein involved in several steps of SARS-CoV-2 life cycle, such as assembly, budding, envelope formation, and pathogenesis (Wu et al., 2020). As the key protein mediating entry to host cells via angiotensin-converting enzyme 2 (ACE2), S protein plays a crucial role in SARS-CoV-2 infection. The structure of the S protein includes S1 (residues 14-685) and S2 (residues 686-1,273) subunits that are separated by a proteolytic cleavage site (Overduin et al., 2022). The S1 subunit binds to the host cell receptor ACE2, whereas the S2 subunit is involved in membrane fusion between the viral and cellular membranes (Huang et al., 2020). S protein comprises three domains: an external domain (residues 1-1,213), a transmembrane domain (residues 1,214-1,234), and a short cytoplasmic tail (residues 1,235-1,273). External domain contains S1 subunit and the majority of S2 subunit. Both transmembrane domain and cytoplasmic tail are in the S2 subunit. The cytoplasmic tail of S protein interacts with host trafficking proteins and subsequently facilitates the cell surface localization of S protein (Figure 1). The palmitoylation of S protein also plays an important role in the life cycle of SARS-CoV-2. Therefore, the understanding of intracellular trafficking and membrane location of S protein may provide mechanistic insights into the treatment or precaution of SARS-CoV-2. In this review, we will summarize how cytoplasmic tail of S protein determines its intracellular trafficking and membrane location.

## COPI

Coatomer protein-I (COPI) is comprised of seven subunits namely α, β, β’, γ, δ, ε and ζ, assembling into trafficking vesicles (Waters et al., 1991). With a cage-like lattice structure, COPI mediates the protein trafficking from the Golgi apparatus back to the endoplasmic reticulum (ER), as well as trafficking within the cisternaes of Golgi apparatus (Hsu et al., 2009). The cargoes of COPI are numerous, such as escaped proteins or lipids. Interestingly, COPI-coated vesicles are involved in the life cycling of multiple viruses (Zhang et al., 2009; Yang and Zhang, 2012; Li et al., 2014; Zhang and Zhang, 2017), including SARS-CoV-2 (Cattin-Ortolá et al., 2021; Jennings et al., 2021; Dey et al., 2022).

After SARS-CoV-2 S protein binds its host receptor ACE2 in the plasma membrane, it entries the target cells through endocytosis, forming a virion endosome encapsulating by a lipid bilayer (Daniloski et al., 2021). In the cytoplasm, the endosomes containing the whole coronavirus virion undergo the membrane fusion and the cleavage of S protein by host proteases (Cattin-Ortolá et al., 2021). Newly synthesized S protein in ER can be transported to Golgi, and then back by COPI vesicle to the endoplasmic reticulum-Golgi intermediate compartment (ERGIC), where the progeny virions assemble. The cytoplasmic tail of S protein in SARS-CoV-2 is crucial for the COPI binding. Researchers have mapped out the COPI binding site in the cytoplasmic tail of S protein (Figure 1). It was the distal half of the cytoplasm tail (residues 1,255-1,273) that bound to COPI (Figure 1) (Cattin-Ortolá et al., 2021). This distal region containing a cytosolic dibasic motif K-X-H-X-X (X can be arbitrary amino acid) was required for the interaction of COPI. COPI binding motif in S protein was sub-optimal due to the His at residue 1,271 (Cattin-Ortolá et al., 2021). The weak interaction between SARS-CoV-2 S protein and COPI attributed to the COPI binding region K-L-H-Y-T, which was different from the canonical binding motif such as K-X-K-X-X or K-K-X-X. Thus, several variants were employed to determine the effect of the binding among these mutant motifs. Eventually they confirmed that the change from H to K in the 1,271 amino acid of S protein increased the binding of COPI, whereas, the K1269A and H1271A mutants reduced the COPI binding (Cattin-Ortolá et al., 2021).

Researchers have examined the effect of S protein C-terminal residues in modulating COPI binding using a biolayer interferometry assay (BLI) assay (Dey et al., 2022). Three diverse mutations of the S protein hepta-peptide, namely acidic (Glu), basic (Arg), and neutral (Gln) amino acid, were employed to test the binding effects that were influenced by the acid-base property of C-terminal residues. Acidic Glu in the C-terminal S protein tended to more likely associate with complementary charged basic residues in COPI. This interaction strengthens the binding between S protein and COPI in host cell, while the basic and neutral mutants did not increase the binding affinity. None of the coronavirus S proteins contained a C-terminal acidic residue within the K-X-H-X-X, K-X-K-X-X, or K-X-R-X X dibasic motif. This result was consistent with the former result focusing on the binding motif rather than the acid-base property and complementary charged function (Dey et al., 2022).

Previous studies have shown that the binding site for host cargo dibasic motifs in COPI complex is located in the N-terminal β-propeller WD40 domains of α and β′COPI subunits, both of which are homologous in structure (Eugster et al., 2004; Jackson et al., 2012). The crystal structure of the depurative αCOPI-WD40 domain was solved to the resolution of 1.8 Å. In 3D structure, the αCOPI-WD40 domain was organized into a β-propeller. Moreover, researchers also identified that there was an N-terminal acetylation of the αCOPI-WD40 polypeptide in the structure. All of these crystal structures were the basis of the sequence specific binding between S protein and αCOPI-WD40 domain. Structural analysis demonstrated that the αCOPI-WD40 domain could interact directly with the S protein due to the hepta-peptide in S protein C-terminal with the corresponding structural information. According to the *in silico* modeling of S protein hepta-peptide, αCOPI-WD40 domain contained predominantly polar residues. Among these residues, Arg57, Asp115, and Tyr139 played significant roles in the stabilization of S protein hepta-peptide binding (Dey et al., 2022). Arg57 in αCOPI-WD40 interacted with His1271, Tyr1272, and Thr1273 in S. Asp115 in αCOPI-WD40 associated with Lys1269 side-chain of S. Moreover, this side-chain of Lys1269 further interacted with the hydroxyl oxygen in Tyr139 side-chain of αCOPI-WD40 domain. Collectively, residues in WD40 domain, including Arg57, Asp115, and Tyr139, offered an extensive and polar interaction network for connection with the amino acid in cytoplasmic tail of S protein involving Lys1269, His1271, Tyr1272 and Thr1273.

To further confirm the function of amino acid residues in αCOPI-WD40 domain, experiments on the mutagenesis of these three αCOPI-WD40 residues to Ala were conducted, aiming to detect the binding efficiency (Dey et al., 2022). These three mutants were analyzed for binding to the wild-type S protein hepta-peptide. Consequently, none of the three mutants indicated any association with the wild-type sequence of the S protein hepta-peptide, which suggested that any of the three resides were respectively indispensable for the binding to the S protein hepta-peptide. Overall, the deletion of even one of these resides might contribute to the destabilized binding between αCOPI-WD40 domain and cytoplasmic tail of S protein.

It should be noted that no matter in bats, pangolins, camels, and humans, which have been implicated as hosts for SARS-CoV-2, Arg57, Asp115, and Tyr139 in αCOPI are identical (Dey et al., 2022). An analysis of αCOPI sequence conservation across 150 species showed that Arg57 and Asp115 were completely conserved, while Tyr139 was replaced by Phe or Trp in 5.3% or 0.7% of the sequences. To some extent, this result suggested an evolutionary conserved sequence on these three residues in binding dibasic motifs of host proteins, which is employed by the sarbecovirus S protein to hijack the host COPI machinery.

In the study of Cattin-Ortolá *et al*, they found that WD40 domain of human β′COP associated with S tail in GST pulldown experiments (Cattin-Ortolá et al., 2021). However, they did not test the interaction between S tail and WD40 domain of αCOP. In the study of Dey *et al*, they examined the interaction between hepta-peptide from S tail and WD40 domain from yeast β′COP or WD40 domain from yeast αCOP in BLI experiments (Dey et al., 2022). They identified the binding of yeast αCOPI-WD40 to hepta-peptide from S tail. However, WD40 domain from yeast β′COP did not associate with hepta-peptide from S tail. Arg57, Asp115, and Tyr139 in WD40 domain in αCOP are the key residues for S binding. It is likely that those three residues in the correlated position of β′COP are also the determinant for S binding. Tyr in human β′COP is replaced by Phe in yeast β′COP, which explains the lack of interaction between S tail and WD40 domain from yeast β′COP in the study of Dey *et al.* Thus, we believe that both αCOPI-WD40 and β′COP-WD40 in human could interact with S tail (Figure 1).

Why SARS-CoV-2 S protein is evolved into having the sub-optimal or weak binding for COPI instead of remaining the canonical motif? It is speculated that this distinct motif seems to facilitate the expression of S protein at the cell surface, in order to interact with adjacently non-infected cells rather than stay in the cytoplasm waiting for the newly assembled virions. Having been located at the plasma membrane, S protein did not contribute to virion formation. However, it mediated the formation of syncytia, a multinucleated cell resulting from multiple cell fusions of uninuclear cells, and then accelerated the infectious progress before the release of progeny virions. Collectively, the COPI binding motif in SARS-CoV-2 S protein could be utilized to transport S protein back to the ERGIC for assembly. Meanwhile, this binding is not perfect, making S protein localize less in the cytoplasm but more on the surface to increase the cell fusions.

## COPII

Both COPI and coat protein complex II (COPII) are responsible for membrane trafficking between the ER and the Golgi. Transport of transmembrane and soluble proteins from the ER to the Golgi apparatus are driven by COPII (Kuehn and Schekman, 1997). Classical COPII is composed of a small GTPase, SAR1, and 2 cytosolic protein complexes, the inner coat Sec23-Sec24 heterodimer and outer coat Sec13-Sec31 heterotetramer (Sato and Nakano, 2007). Generally, cargoes are incorporated into COPII vesicles through either “cargo capture” or “bulk flow”. The former mechanism utilizes receptor-mediated ER export of proteins to enter COPII vesicles, while the bulk flow is through passive diffusion (Sato and Nakano, 2007).

Interestingly, S protein of SARS-CoV-2 had been proven to be the cargo of COPII. Since the trafficking of membrane proteins through the secretory pathway is conventionally involved in the interactions between cytoplasmic tails of membrane proteins and coat proteins in the cell, researchers conducted further experiments to map out the COPII binding site in the cytoplasmic tail of S protein (Figure 1). They generated several mutations in the residues of cytoplasmic tail of S. COPII binding of S protein located in the acidic residues DEDDSE, containing the di-acidic ER exit motif, which can be recognized by the Sec24 subunit of the COPII (Cattin-Ortolá et al., 2021). Having determined the binding site for COPII, researchers subsequently examined the function of this binding site. In the mutation of the acidic residues of COPII binding region, cell surface expression of S protein was decreased significantly. Meanwhile, mutated S protein accumulated in the ER. These results indicated that the residues DEDDSE directed efficient egress of the newly synthesized S protein out of the ER and eventually on the plasma membrane.

## SNX27

As a member of the sorting nexin family of proteins, sorting nexin 27 (SNX27) encompasses three structural domains, respectively, a central phox homology (PX) domain, an amino-terminal postsynaptic density protein 95 (PSD-95), disks large, and zona occludens (PDZ) domain, and a carboxyl-terminal 4.1/ezrin/radixin/moesin (FERM) domain (Lu et al., 2022). SNX27 is an endosome-associated cargo adaptor protein, which mediates endocytic recycling of cargo proteins from endosomes to the plasma membrane, meanwhile preventing the lysosomal degradation (Lu et al., 2022). SNX27 is involved in the trafficking of over 400 receptors, transporters, channels, enzymes, and adhesion molecules, including the angiotensin-converting enzyme 2 (ACE2), glucose transporter GLUT1, numerous G-protein-coupled receptors (GPCRs) such as the β2 adrenergic receptor (β2AR) and parathyroid hormone receptor (PTHR) (Chandra et al., 2021).

For SARS-CoV-2, SNX27 interacts with its S protein to facilitate its surface expression and thus accelerate the infectious process. After the membrane fusion and cleavage of virion in the cytosol, full length of the S protein is exposed to the cytoplasm (Cattin-Ortolá et al., 2021). Therefore, SNX27 could interact with the dissociative S, and mediate endocytic trafficking of S protein. Since SNX27 is responsible for endosome-to-plasma membrane recycling of membrane proteins, some researchers tended to explore its precise function of S. They found, at the beginning of endocytosis, depletion of SNX27 did not reduce the plasma membrane surface expression of S. Whereas, the deletion of SNX27 contributed to the decreasing colocalization of S protein with early endosomal marker, but the increasing colocalization of S protein with late endosomal marker (Zhao et al., 2021). Consequently, these results identified that SNX27 might promote endocytic recycling of S protein and prevent it from the lysosomal degradation pathway.

Further experiments were performed to dissect which domain of SNX27 could interact with S protein. Co-immunoprecipitation (Co-IP) assay showed that deletion of the PDZ domain, but not PX or FERM domain abrogated the interaction between SNX27 and S, indicating that the PDZ domain of SNX27 is required for this binding. Moreover, it was also found that the mere PDZ domain of SNX27 was sufficient to bind to S protein. Generally, PDZ domain can engage with both retromer and PDZ binding motifs at the C-terminus of transmembrane proteins, mediating their endocytic trafficking and recycling (Steinberg et al., 2013). Retromer, considered as “master regulator” of protein sorting at endosomal membranes, is a heterotrimer formed from vacuolar protein sorting protein 35 (Vps35), Vps29, and Vps26 (Lu et al., 2022). And Vps26 associates with the PDZ domain of SNX27 (Lucas et al., 2016). As one of the best characterized endosomal retrieval complexes, retromer is responsible for identifying specific signals in the cytoplasmic domains of cargo proteins, thereby preventing from the endosomal degradation pathway (Yong et al., 2022). SNX27-retromer complex selectively recycles numerous cargoes exclusively to the plasma membrane and is not required for endosome-to-trans-Golgi-network retrieval (Yong et al., 2022). Hence, researchers examined whether the connection between SNX27 and S protein relies on retromer. A SNX27 mutant deficient for retromer binding was generated. In this mutant, SNX27 retained the ability to bind S protein, indicating that SNX27 associates with S protein independent of retromer (Zhao et al., 2021).

As most cargoes utilize PDZ binding motif (PBM) to interact with SNX27, does S protein exploits the same PBM to bind SNX27 as other cargoes canonically? Intriguingly, S protein did not contain a PBM. The binding site in S protein was in the “MTSC” motif at the cytoplasmic tail of S protein (Zhao et al., 2021). This “MTSC” motif was in the N-terminal half of the tail nearest the transmembrane domain of S protein (Figure 1). Also, this region contained abundant cysteines. Furthermore, immunoprecipitation experiments showed that mutations of either MT or SC residues significantly reduced the binding of S protein to SNX27-PDZ.

## Palmitoylation

Palmitoylation is a crucial form of lipid posttranslational modification, which could add the palmitic acid (PA), a 16-carbon saturated fatty acid, to the thiol group of a cycteine residue of substrate. Palmitoylation influences the function of proteins by regulating their transport, stability, and localization (Main and Fuller, 2022). In particular, it increases protein hydrophobicity and facilitates protein trafficking to cellular membranes (Zhang et al., 2019; Main and Fuller, 2022).

C-terminal cytoplasmic tail of SARS-CoV-2 S protein is consisted of 9 cysteine residues on the proximal side of the membrane (C1235, C1236, C1240, C1243, C1247, C1248, C1250, C1253, and C1254), while the N terminus of SARS-CoV-2 S protein contains a single Cys sites (C15). With such abundant cysteine sequences, S protein is palmitoylated (Mesquita et al., 2021; Wu et al., 2021; Li et al., 2022). In order to investigate which cysteine residues are the palmitoylation sites of S protein, researchers generated three mutants, C15S (C1), C1235S/C1236S/C1240S/C1243S/C1247S/C1248S/C1250S/C1253S/C1254S (C9), and C15S/C1235S/C1236S/C1240S/C1243S/C1247S/C1248S/C1250S/C1253S/C1254S (C10) (Li et al., 2022). Through the acyl-biotin exchange (ABE) assay, researchers found that both C1 and C9 led to a reduced palmitoylation signal. Consistently, C10 mutant, in which all 10 cysteines were replaced to serine, showed the least palmitoylation signal, indicating that the N-terminal C15 and C-terminal cytoplasmic tail were the two crucial sites for the palmitoylation of S protein (Figure 1) (Li et al., 2022). However, as they did not perform the single point mutation, they couldn’t draw the conclusion that all of the cysteines in the cytoplasmic tail are required for S protein palmitoylation. While, in another study, researchers changed group of 2-3 adjacent cysteines to alanine, creating four spike protein mutants (Mesquita et al., 2021). All of the four mutants led to a reduced level of palmitoylation. Especially, one of the mutants, where the two most membrane-proximal cysteines were replaced to alanine, caused up to 80% drop. Thus, it can be concluded that all of 10 cysteines are involved in the palmitoylation of S protein, and the cysteines adjacent to the membrane play a predominant role (Mesquita et al., 2021). Moreover, they also measured the kinetics of palmitoylation. Compared with rapid plamitoylation of wide type S protein, two of the mutants, in which the membrane-proximal cysteines were changed, showed a slow and limited palmitoylation. However, the other two mutants had no major effect on plamitoylation kinetices, inferring that palmitoylation of the cysteine residues proximal to the membrane of S protein could facilitate palmitoylation process of distal cysteine residues (Mesquita et al., 2021).

Palmitoylation is catalyzed by a family of 23 palmitoyltransferases containing Zinc finger Asp-His-His-Cys (DHHC) domain (ZDHHCs) (Zaballa and van der Goot, 2018). ZDHHC family is consisted of ZDHHC1 to 24, and ZDHHC10 doesn’t exist in human. To identify putative members of the ZDHHC family proteins that palmitoylate S protein, reserachers individually transfected with 23 ZDHHCs into 293T cells and performed Co-IP experiments (Li et al., 2022). They monitored the plamitoylation level of S protein after the overexpression of these ZDHHCs. ZDHHC2, ZDHHC3, ZDHHC4, ZDHHC5, ZDHHC8, ZDHHC9, ZDHHC11, ZDHHC14, ZDHHC16, ZDHHC19, and ZDHHC20 could promote the palmitoylation of S protein (Li et al., 2022). In another study, researchers screened all 23 ZDHHCs and found that ZDHHC2, ZDHHC3, ZDHHC6, ZDHHC11, ZDHHC12, ZDHHC20, ZDHHC21, and ZDHHC24 could plamitoylate S protein (Puthenveetil et al., 2021). The fatty acid synthase inhibitor C75 and palmitate analog 2-bromopalmitate (2-BP), which is commonly used to inhibit palmitoylation, were utilized to further determine the significance of ZDHHCs in S protein palmitoylation (Li et al., 2022). Both C75 and 2-BP could reduce the palmitoylation of S protein, confirming the palmitoylation of SARS-CoV-2 S protein. Ramadan AA *et al* found that 2-BP could also impair the SARS-CoV-2 cell fusion and virus infectivity (Ramadan et al., 2022). Among all of the ZDHHCs, Mesquita FS *et al* individually depleted all ZDHHC enzymes. Downregulation of ZDHHC8, ZDHHC9 and ZDHHC 20 contributed to a more than 50% decrease in the palmitoylation level of S protein (Mesquita et al., 2021).

For enveloped viruses, cholesterol is an important determinant of local membrane curvature in viral membrane fusion and surface glycoprotein regulation. The binding of cholesterol and S protein plays a crucial role in SARS-CoV-2 infection. It is known that wild type S protein could be palmitated and targeted to cholesterol-rich membrane microdomains. While, Cys-less (ΔCys) form of S protein was compromised in its ability to incorporate into virions, possibly due to the failure of palmitoylation and leading to the improper translocation instead of to cholesterol-rich domains (Puthenveetil et al., 2021). Furthermore, Li *et al* investigated the concrete function of palmitoylation of S protein. For plasma membrane targeting, they found there was no significant distinction between S-WT or C10 mutant in the level of plasma membrane portion, suggesting that palmitoylation is not required for membrane location (Li et al., 2022). Next, they investigated the role that palmitoylation played on the viral entry and syncytia formation. C1, C9 and C10 mutants showed the reduced entry abilities and SARS-CoV-2 S-mediated syncytia formations became fewer in these three mutants as well (Li et al., 2022). Nevertheless, the specific mechanism of S protein palmitoylation-mediated syncytia formation has not been elucidated in detail and further research is demanded in the future. Another study revealed that palmitoylation of S could facilitate its biosynthesis, by reducing the turnover rate of fully folded S protein and increasing the flux of newly synthesized S protein through ER quality control (Mesquita et al., 2021). Additionally, the newly synthesized S protein would undergo degradation without palmitoylation, suggesting that this modification could protect S protein. Meanwhile, palmitoylation seemed to enhance the assembly of ordered lipid domains in the ERGIC, thereby regulating the formation of the viral envelope lipid bilayer (Mesquita et al., 2021). These findings provided novel concepts for studying palmitoylation and lipid biosynthesis of coronaviruses and other enveloped viruses.

## Conclusion and prospect

**FIGURE 2:**
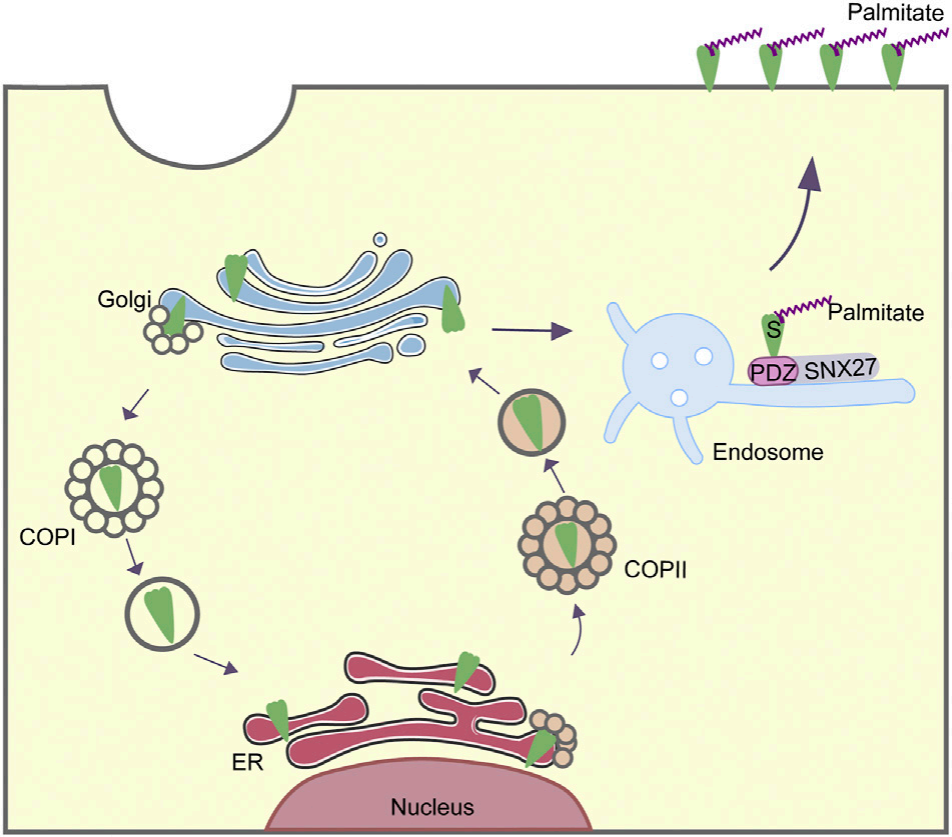
Intracellular trafficking of SARS-CoV-2 S protein mediated by COPI, COPII and SNX27.

The cytoplasmic tail of SARS-CoV-2 S protein directly associates with transport proteins, thus mediates intracellular trafficking and membrane location of S protein. In the distal half of S protein cytoplasm tail, the cytosolic dibasic motif composed of K-X-H-X-X could interact with COPI, which was responsible for retrograde trafficking from Golgi apparatus back to ER. Having bond to COPI, S protein would be transported back to ER, in order to make preparations for the assembly of new virions (Figure 2). However, this binding was sub-optimal, which could facilitate the surface expression of S protein as well as promote the syncytia formation. S protein also interacted with SNX27. With residues “MTSC” next to the transmembrane domain, S protein could be transported into the surface of host cell by SNX27 and retromer. Thus, S protein located more on the surface, hence infected adjacent cells without the release of newly synthesized viruses. COPII is another transport pathway involved in S protein trafficking (Figure 2). COPII mediated the egress of the newly generated S protein from the ER to the secretory pathway. A single palmitoylation site (C15) at N terminus and nine Cys sites within cytosolic C-terminus domain of S protein were confirmed to be the key palmitoylation sites, which could be catalyzed by a family of zinc finger DHHC domain-containing protein palmitoyltransferases (ZDHHC).

The role of SNX27 in the trafficking of S protein is similar to the trafficking of other cargoes, thus implying the competitive inhibition between S protein and conventional cargoes of SNX27. Usually, SNX27 takes charge of the endocytic recycling of cargoes to localize on the plasma membrane, with the assistance of retromer. Since the S protein hijacked SNX27, S protein was more likely to prevent the endocytic trafficking of traditional cargoes of SNX27, resulting in their lysosomal degradations. As the normal cargo sorting function of SNX27 was inhibited, numerous human physiology conditions relying on SNX27 including hypertension, diabetes, neuronal regeneration, copper transport and glucose transport would be disrupted (Chandra et al., 2021). This downregulation of SNX27 cargoes tended to cause a number of Long-COVID symptoms. One of the prominent cargoes transported by SNX27 was ACE2, a receptor that acted as the negative regulator of the renin-angiotensin system. ACE2 has multiple functions such as anti-oxidative stress, anti-inflammatory properties and vasorelaxation (Turner and Nalivaeva, 2022). Based on GST pulldown experiments, with the existence of S protein, ACE2 showed the reduced binding capability to SNX27, as well as decreased surface level (Ren et al., 2022b). Hence, S protein could compete with ACE2 for associating with SNX27 (Ren et al., 2022b). S protein blocked SNX27-ACE2 interaction through its interaction with SNX27, suppressed the endocytic recycling of ACE2 through SNX27, and thus contributed to the reduction of ACE2 in the membrane surface. With low amount of ACE2 in surface, its physiological function would be disrupted. The deficiency of ACE2 may lead to various diseases including lung injury, hypertension, diabetes, abnormal coagulation and so on. Interestingly, these symptoms show significant overlap of Long-COVID symptoms. Thus, it is speculated that Long-COVID may have some potential association with the reduction of ACE2 due to S protein. S protein, which would need time to be degraded, may lead to the Long-COVID. Besides, the inhibition of endocytic recycling of GLUT1 might be another reason for side effects on nervous system. GLUT1 facilitated the glucose supply to the brain and other organs (Tang and Monani, 2021). S protein suppressed the association between GLUT1 and SNX27, preventing its endocytic recycling in a similar way as the inhibition to ACE2 (Ren et al., 2022a). In the patients recovered from acute symptoms, the remaining of S protein contributed to GLUT1-deficiency, which may explain the neurological symptoms in the Long-COVID, such as seizures and persistent or paroxysmal neuromuscular disorders. However, the evidences supporting the impact of ACE2-reduction and GLUT1-reduction on Long-COVID seems to be not abundant, and this inference needs to be further confirmed through more experiments.

Since S protein could interfere with SNX27-mediated endocytic recycling, we speculate that there may be several pharmaceutical and therapeutic options for treating the Long-COVID. Firstly, antibodies against S protein specifically may be a suitable choice. Moreover, enzymes that degrade S protein could be applied to treat Long-COVID. Additionally, we can impair the function of S protein at the transcriptional level. By generating siRNA against SARS-CoV-2 S, the expression of S protein would be decreased.

As plamitoylation is crucial for S-mediated syncytia formation and SARS-COV-2 entry, there are many other membrane proteins that need to be palmitoylated to perform their functions as well. We speculate that S protein might hijack ZDHHCs and therefore inhibit the palmitoylation of other proteins in host cell. For example, ACE2 would be palmitoylated before the formation of extracellular vesicles, which was also catalyzed by ZDHHC (Xie et al., 2021). It is possible that S protein could inhibit membrane location of ACE2. Less ACE2 receptor on membrane might impair its physiological function, which was likely to be another cause of Long-COVID.
